# Ex vivo/in vitro protective effect of myricetin bulk and nano-forms on PhIP-induced DNA damage in lymphocytes from healthy individuals and pre-cancerous MGUS patients

**DOI:** 10.1007/s00204-020-02754-x

**Published:** 2020-04-27

**Authors:** Shabana Akhtar, Mojgan Najafzadeh, Mohammad Isreb, Lisa Newton, Rajendran C. Gopalan, Diana Anderson

**Affiliations:** 1 School of Chemistry and Biosciences, University of Bradford, Richmond Building, Bradford, BD7 1DP UK; 2 School of Pharmacy and Medical Sciences, University of Bradford, Bradford, UK; 3 Bradford Royal Infirmary (BRI), Bradford, UK

**Keywords:** Myricetin, Bulk and nano-forms, Pre-cancerous patients, Lymphocytes, PhIP, P53, ATR

## Abstract

2-Amino-1-methyl-6-phenylimidazo [4,5-*b*]pyridine (PhIP) is a central dietary mutagen, produced when proteinaceous food is heated at very high temperatures potentially causing DNA strand breaks. This study investigates the protective potential of a well-researched flavonoid, myricetin in its bulk and nano-forms against oxidative stress induced ex vivo*/*in vitro by PhIP in lymphocytes from pre-cancerous monoclonal gammopathy of undetermined significance (MGUS) patients and those from healthy individuals. The results from the Comet assay revealed that in the presence of myricetin bulk (10 µM) and myricetin nano (20 µM), the DNA damage caused by a high dose of PhIP (100 µM) was significantly (*P* < 0.001) reduced in both groups. However, nano has shown better protection in lymphocytes from pre-cancerous patients. Consistent results were obtained from the micronucleus assay where micronuclei frequency in binucleated cells significantly decreased upon supplementing PhIP with myricetin bulk (*P* < 0.01) and myricetin nano (*P* < 0.001), compared to the PhIP treatment alone. To briefly determine the cellular pathways involved in the protective role of myricetin against PhIP, we studied gene expression of P53 and ATR kinase (ATM- and Rad3-related), using the real-time PCR technique.

## Introduction

Recently, the consumption of processed and overcooked red meat has been associated with causing carcinogenicity in humans (Bouvard et al. 2015), attributed to the production of food-related carcinogens including heterocyclic amines (HCAs) (Sugimura et al. 2004). HCAs are strong DNA-damaging complexes which are formed when meat and other related products are cooked at very high temperature (Turesky and Le Marchand 2011). 2-amino-1-methyl-6-phenylimidazo [4,5-*b*] pyridine (PhIP) is considered as the most commonly occurring HCA in our diet (Sugimura et al. 2004). It is metabolically activated by cytochrome P450 1A2 (CYP1A2) producing the toxic intermediate, 2-hydroxyamino-1-methyl-6-phenylimidazo [4,5-*b*] pyridine (N–OH–PhIP) (Turesky 2007). It has been stated that increasing PhIP doses cause cellular death while surviving cells exhibit high levels of mutations, as determined in the hypoxanthine–guanine phosphoribosyl transferase *(hprt)* locus (Gooderham et al. 2002). Several in vivo (Cheung et al. 2011; Choudhary et al. 2012; Li et al. 2012) and epidemiological studies (Cross et al. 2005; Voutsinas et al. 2013) reported the contribution of PhIP towards the induction of mammary, gastrointestinal and prostate cancers in rodents. C8-PhIP-dG adducts are said to interfere with the DNA replication process triggering a cellular stress response called replication stress mainly through DNA damage which ultimately gives rise to single stranded DNA (ssDNA) **(**Byun et al. 2005). The ssDNA quickly forms an ssDNA-RPA complex by adjoining with the replication protein A (RPA) which is then detected by ATR-interacting protein (ATRIP) recruiting kinase ATM- and Rad3-related (ATR). Thus, this process triggers a DNA damage response (DDR) by activating ATR (Zou and Elledges, 2003; Ball et al. 2005). ATR in cooperation with RPA, therefore, plays a significant role in induction of the repair pathways and facilitates the restart of hindered replication forks by stabilizing them (Cimprich and Cortez 2008).

Myricetin, a well-studied flavonoid with diverse properties is primarily recognised due to its anti-oxidant, anti-cancer and anti-inflammatory activities (Ong and Khoo 1997). It has also been regarded as a potent chemo preventative agent against various tumours. Myricetin is a plant-derived flavonoid, mainly occurring in tea, berries, red wine, fruits and vegetables (Androutsopoulos et al. 2009; Kim et al. 2014; Semwal et al. 2016). Myricetin displayed anti-genotoxic effects against the food mutagens, 3-amino-1-methyl-5H-pyrido-(4,3-b) indole (Trp) and 2-amino-3-methylimidazo-(4,5-f) quinoline (IQ) and decreased DNA damage without exogenous metabolic initiation in human lymphocyte cells (Anderson et al. 1997).

Diet is an important contributory factor towards the development of various cancers and knowing that lymphocytes express CYP1A2 and that the food mutagen, PhIP, activated by CYP1A2, contributes in many dietary tumours (Anderson et al. 1997; Cheung et al. 2011; Voutsinas et al. 2013), we investigated for the first time in this study the effects of PhIP at basal levels as well as by co-supplementation with either myricetin bulk (MYR B) or myricetin nano (MYR N) in lymphocytes from healthy individuals and pre-cancerous, monoclonal gammopathy of unknown significance (MGUS) patients.

In this study, we investigated and analysed the effects of PhIP treatment on the induction of DNA damage, strand breaks formation using the Comet plus cytogenetic damage in the micronucleus assays, kinase ATR regulation and p53 levels using real-time PCR in peripheral lymphocytes from pre-cancerous patients and healthy individuals and also the modulating effects of myricetin (MYR B and MYR N) on PhIP-induced metabolic changes of these factors.

## Methodology

### Blood sample collection and Ethics

The current study which involved the use of human peripheral lymphocytes has been granted ethical approval by Leeds East Ethics Committee (Reference No. 12/YH/0464) and the University of Bradford’s Sub-Committee for Ethics in Research involving healthy Human Subjects (Reference No. 0405/8). The research support and governance office of Bradford Teaching Hospitals NHS Foundation also agreed the research number (REDA 1202).

The blood samples from healthy individuals and pre-cancerous patients used in the study were collected after obtaining informed consent from volunteers and are listed in Tables 1 and 2, respectively.

**Table 1:** Characteristics of healthy blood samples

No	Age	Ethnicity	Gender	Smoking history	Family history
1	25	Caucasian	M	No	None
2	44	Asian	M	Yes	None
3	28	Caucasian	M	No	None
4	23	Caucasian	F	No	None
5	27	Caucasian	M	No	None
6	33	Arab	M	Yes	None
7	47	Asian	M	Yes	None
8	28	Caucasian	M	No	None
9	42	Asian	M	No	None
10	48	Asian	M	No	None
11	60	Asian	M	Yes	None
12	24	Asian	M	No	None
13	34	Asian	M	No	None
14	34	Caucasian	F	Yes	None
15	34	Asian	M	No	None
16	59	Caucasian	F	Yes	None
17	28	Asian	M	Yes	None
18	61	Caucasian	F	No	None
19	36	Caucasian	F	No	None
20	52	Caucasian	F	No	None

*M* male, *F* female

**Table 2:** Characteristics of pre-cancerous patients’ blood samples

No.	Age	Ethnicity	Gender	Smoking history	Family history	Medical condition
1	63	Caucasian	M	Yes	None	MGUS
2	75	Caucasian	M	No	None	MGUS
3	74	Caucasian	F	No	Lung cancer	MGUS
4	83	Caucasian	M	No	None	MGUS
5	60	Asian	F	No	None	MGUS
6	62	Caucasian	M	No	None	MGUS
7	51	Caucasian	F	No	None	MGUS
8	80	Caucasian	M	No	Cancer positive	MGUS
9	81	Caucasian	F	No	Bowel and stomach	MGUS
10	63	Caucasian	M	Yes	None	MGUS
11	63	Caucasian	M	Yes	None	MGUS
12	74	Caucasian	M	No	None	MGUS COPD
13	63	Caucasian	F	Yes	Arthritis	MGUS, COPD
14	66	Caucasian	F	No	Breast cancer	MGUS
15	52	Caucasian	M	Yes	None	MGUS
16	79	Caucasian	M	No	None	MGUS
17	80	Caucasian	F	No	None	MGUS
18	78	caucasian	M	No	None	MGUS
19	50	Asian	F	No	None	MGUS
20	69	Caucasian	M	No	Stomach and lung	MGUS

*M* male, *F* Female, *MGUS* monoclonal gammopathy of unknown significance, *COPD* chronic obstructive pulmonary disease

### Cell culture

Isolated lymphocytes from healthy individuals and pre-cancerous patients were maintained in RPMI medium supplemented with 15% foetal bovine serum (FBS) and 1% penicillin streptomycin (all from Invitrogen, UK) at 37 ℃ to be used in subsequent experiments. However, fresh blood samples were diluted in a 1:1 ratio with RPMI-1640 medium supplemented with 10% DMSO (Invitrogen, UK) and aliquoted volumes were immediately stored at − 80 ℃ to be used in the Comet assay.

### Preparation and concentration of PhIP and myricetin

In this study two forms of myricetin (NP and bulk) and one form of PhIP (Toronto Research chemicals INC, Canada. A617000) (bulk) was used. Myricetin powder (> 96% purity) was purchased from Fisher Scientific, UK. Suspensions of myricetin bulk, myricetin nano and PhIP were made in an excipient mixture (containing 7% (w/w) solid loads of myricetin in a medium comprising of hydroxypropyl methylcellulose (HPMC) (0.5% w/w), sodium lauryl sulphate (SLS) (0.1% w/w), ethanol (0.8% w/w), polyvinylpyrrolidone (PVP) K-30 (0.5% w/w) and purified water). The suspensions were stored in amber glass bottles at 4 ℃ for the research duration. The concentrations of myricetin bulk (MYR B) and NP (MYR N) forms, used for this research study were 10 µM and 20 µM, respectively (data used from previous study) (Akhtar et al. 2020). The concentration of PhIP used throughout the study was 100 µM. These values were determined by concentration response curves for each of the chemicals. The stability of the particles for all the chemicals was assessed by checking their particle size and the difference was less than 1%. Hence, these were considered stable to be used for the study. The suspensions were also sonicated for 10 min before each use to avoid sedimentation and control aggregation.

### Cell viability

Lymphocyte viability and integrity were measured using trypan blue exclusion. Cells were isolated and treated with chemicals for 24 h supplemented with complete medium (RPMI media containing 1% penicillin–streptomycin and 15% foetal bovine serum (FBS)) (all from Invitrogen, UK). After the incubation time was over, 10 µl of suspension was added to 10 µl of Trypan blue 4% (Sigma Aldrich, UK) and transferred to a haemocytometer for cell counting to determine viable cells.

### The Comet assay

Lymphocytes were treated with MYR B and MYR N in combination with PhIP (100 µM) for 1 h. The cell suspension was centrifuged at 3000 rpm (1000*g*). The supernatant was removed, and the pelleted cells were used for the Comet assay as previously defined (Tice et al 2000; OECD 2019; Anderson et al. 2014; Azqueta and Dusinska 2015).

### Micronucleus (MN) assay

Fresh blood samples from five healthy and five patients supplemented with Phytohaemagglutinin (PHA) (Invitrogen ltd, UK) were added to conical flasks containing basic culture medium (RPMI 1640 with 25 mM HEPES and l-Glutamine), 1% penicillin–streptomycin and 15% FBS were incubated for 24 h at 37 °C in the presence of 5% CO_2_. The assay procedure was adapted as described by Fenech (OECD 2019; Fenech 2007). To determine the frequency of MNi, 1000 cells were scored according to criteria characterized by Fenech (2007).

### Real-time PCR analysis

Isolated lymphocytes were seeded in six-well plates and treated with chemicals for 24 h. Two micrograms of total isolated RNA was subjected to reverse transcription using iScript™ c DNA synthesis kit (Bio Rad, UK) according to the manufacturer protocol. Each real-time PCR experiment was done thrice in a total of 10 µl reaction mixtures. Data were analysed using the 2-DDCt method (Livak and Schmittgen 2001) and normalised against the internal home gene GAPDH in each sample.

### Statistical analysis

All the experiments were conducted at least three times. Graph Pad prism 7 was used to perform statistical analysis. The results were analysed using one-way analysis of variance (ANOVA) and two-way ANOVA to test differences between each treatment and the control. A *P* value of < 0.05 was considered statistically significant.

## Results

### Concentration response curve for PhIP

**Fig. 1:**
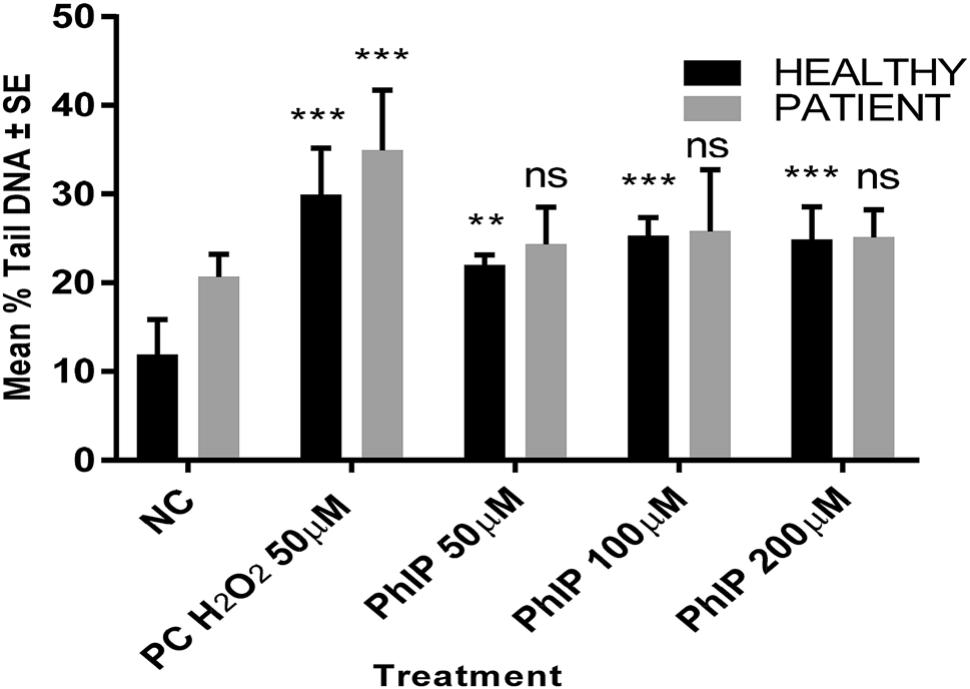
Concentration dependent responses of PhIP in lymphocytes from healthy individuals and pre-cancerous patient lymphocyte showing mean %Tail DNA. All PhIP doses induced genotoxic damage to the cells whereas 100 µM seemed to produce maximum DNA damage in both groups. All data have been expressed as mean ± standard errors (SE). *** *P* < 0.001, ** *P* < *P* < 0.01, *ns* not significant

The optimal dose of PhIP inducing maximum DNA damage was determined using the Comet assay in lymphocytes from healthy vs patient group. PhIP concentrations (50–200 µM) were considered for the test by comparing against the untreated group. 50 µM hydrogen peroxide (H_2_O_2_) was used as a standard positive control. Results demonstrate that all doses of PhIP have induced significant (*P* < 0.001) DNA damage in healthy lymphocytes (Fig. 1). Although there was little difference between the three concentrations (50, 100 and 200 µM), 100 µM was used as a standard which caused maximum DNA damage in both groups. Hence, it was used throughout the study.

### Viability of lymphocytes

**Fig. 2:**
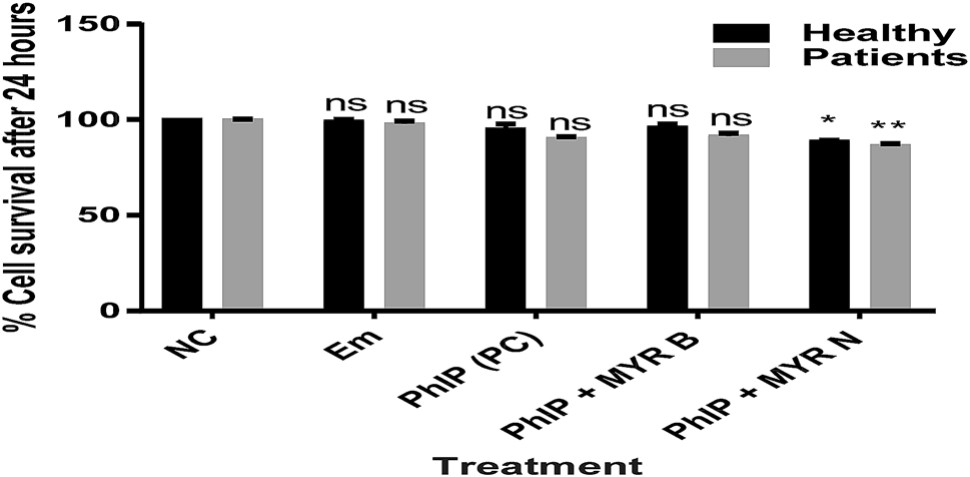
% viability per 100 cells counted/treatment after 24 h. Treatment groups used were untreated (NC), excipient mixture (Em), positive control (PC) PhIP 100 µM, PhIP supplemented with MYR B (10 µM) and MYR N (20 µM). Viability was calculated as more than 80% for all the treatment groups. (*ns* not significant, *P < 0.02, ** *P* < *P* < 0.004)

There was no significant effect observed on viability of lymphocytes from healthy individuals and those from pre-cancerous patients after 24 h treatment with various treatment groups used in this study except for PhIP + MYR N. However, viability for this group was also assessed at more than 86% in both healthy and patient lymphocytes (Fig. 2). This confirms that the concentration of chemicals used throughout the study were non-toxic for the lymphocyte cells.

### Modulating effects of MYR B and MYR N on PhIP-induced DNA damage in lymphocytes using the Comet assay

**Fig. 3:**
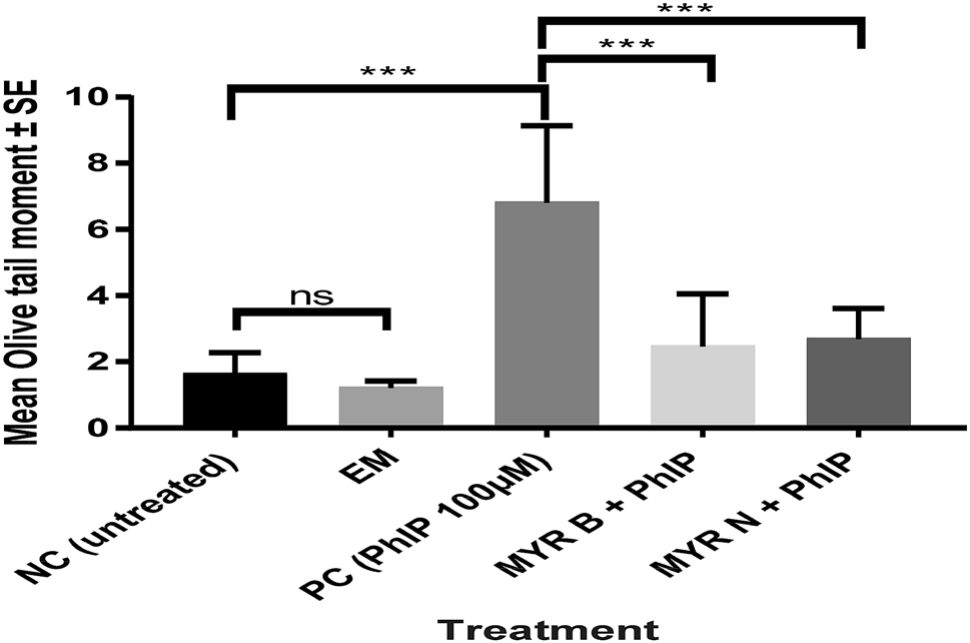
Modulating effect of MYR B & N on PhIP-induced DNA damage in healthy lymphocytes using Olive Tail Moment. The above figure shows five treatment groups including an untreated group, the positive control (PhIP 100 µM), MYR B (10 µM) with PhIP, MYR N (20 µM) supplemented with PhIP and excipient mixture (EM) (0.1%). The PC and EM were compared against the NC while MYR B and MYR N against the PC. The mean NC and PC values for healthy groups were 1 and 6 respectively. ***Represents *P* < 0.001, *ns* not significant. The horizontal lines on top of the graph show the significant difference between the treatment groups

**Fig. 4:**
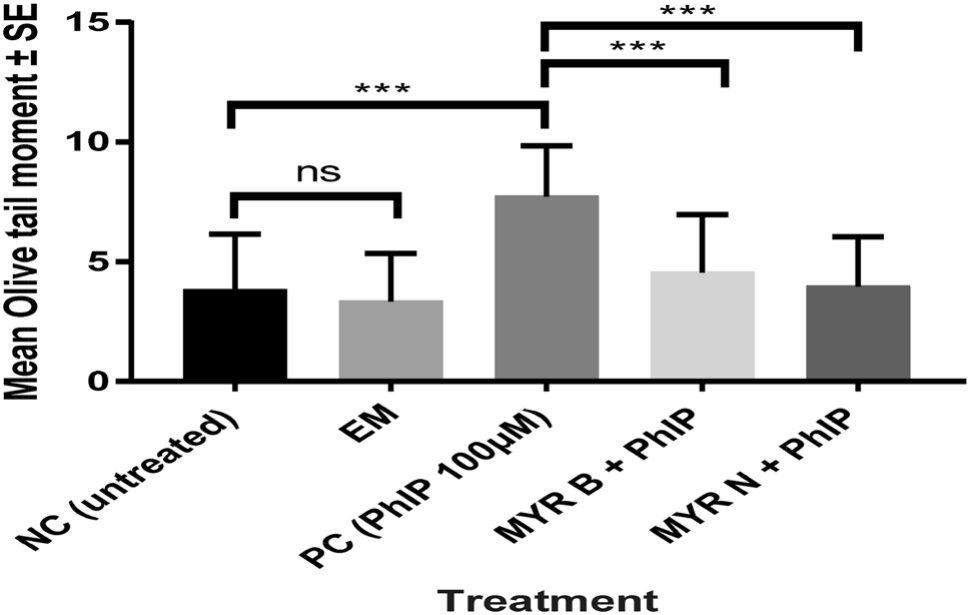
Modulating effect of MYR B and N on PhIP-induced DNA damage in pre-cancerous patient lymphocytes using Olive Tail Moment. The above figure shows five treatment groups including an untreated group, the positive control (PhIP 100 µM), MYR B (10 µM) with PhIP, MYR N (20 µM) supplemented with PhIP and excipient mixture (EM) (0.1%). The PC and EM were compared against the NC while MYR B and MYR N against the PC. The mean NC and PC values for healthy groups were 3 and 7, respectively. *** *P* < 0.001, *ns* = not significant. The horizontal lines on top of the graph show the significant difference between the treatment groups

To determine the in vitro effects of different particle sizes of myricetin on PhIP-induced DNA damage, lymphocytes from healthy volunteers and pre-cancerous patients were treated with either MYR B (10 µM) or MYR N (20 µM) simultaneously co-supplemented with PhIP (100 µM). Results demonstrated a reduction in DNA damage overall, when compared against the PC (PhIP 100 µM). The damage was significantly decreased by both forms of myricetin in healthy lymphocytes as well as in those from pre-cancerous patients assessed using the two parameters of the Comet assay, Olive tail moment (OTM) and % Tail DNA. However, only OTM (Figs. 3, 4) data have been showed because of similar results. The levels were almost returning to those similar to the negative control. This indicates that MYR B and MYR N exhibit similar protective and anti-genotoxic effects and can potentially protect the lymphocytes of healthy individuals and pre-cancerous patients against the DNA damage and genotoxicity caused by PhIP.

### Determination of micronuclei (MNi)

#### MNi frequency in binucleated cells (BiNC)

The effect of PhIP alone and combination of PhIP with MYR B or MYR N on micronuclei formation was assessed using the micronucleus assay. Our results show that the number of MNi in BiNC cells from pre-cancerous patients was higher than those from healthy individuals in their respective untreated groups. There were few MNi observed in the healthy NC group per 1000 cells counted, and this needs to be taken into consideration when evaluating these results. This frequency was further enhanced by exposing the cells to PhIP (100 µM) (*P* < 0.001). However, simultaneous addition of MYR B or MYR N has significantly reduced MNi formation induced by PhIP in BiNC cells from healthy individuals and pre-cancerous patients (Table 3).

**Table 3:** The average of various markers/parameters of chromosomal damage in the cytokinesis-block micronucleus assay

Subject	Treatment Group	NDI	% BiNC	MNi in MoNC	MNi in BiNC
Healthy individuals	NC	1.85	62	0	0
	PhIP (PC)	1.63 (ns)	62 (ns)	7 (*P* < 0.001)	15 (*P* < 0.001)
	MYR B + PhIP	1.83 (ns)	61 (ns)	4 (*P* < 0.01)	10 (*P* < 0.01)
	MYR N + PhIP	1.63 (ns)	61 (ns)	3 (*P* < 0.01)	8 (*P* < 0.001)
Pre-cancerous patients	NC	1.81	61	6	6
	PhIP (PC)	1.78 (ns)	61 (ns)	12 (*P* < 0.001)	13 (*P* < 0.001)
	MYR B + PhIP	1.73 (ns)	60 (ns)	8 (*P* < 0.01)	9 (*P* < 0.01)
	MYR N + PhIP	1.80 (ns)	60 (ns)	7 (*P* < 0.01)	6 (*P* < 0.001)

Showing NDI per treatments on healthy and patient cells (all values compared against respective untreated group), mean % of BiNC (all values compared against respective untreated group), mean number of MNI in BiNC and MNi frequency in MoNC (For these two columns, the PC is compared against the respective NC (untreated lymphocytes). However, co-supplemented groups (MYR B + PhIP, MYR N + PhIP) were compared against their respective PC)

#### Other elements of MN

Table 3 shows that there is no significant difference between the NDI and % of BiNC for all treatment groups when compared to the respective untreated group for healthy individuals and pre-cancerous patients. PhIP significantly induced MNi formation in both groups. Untreated cultures of patient groups have shown higher numbers of MNi both in MoNC and BiNC as compared to the groups treated with MYR B and MYR N. MYR B (10 µM) or MYR N (20 µM) addition with PhIP has significantly reduced the MNi induction regardless of group difference and cell type (Table 3).

**Table 4:** The effect of confounding factors

Subject	Treatment Group	Smoker	Non-smoker	Asian	Caucasian	Male	Female	Young	Old
		OTM	OTM	OTM	OTM	OTM	OTM	OTM	OTM
Healthy individuals	NC	1.1	0.7 ns	0.7	0.7 ns	1.2	0.8 ns	2.7	4.1 ns
	PC (PhIP 50 µM)	9.1	10.7 ns	10.4	9.2 ns	8.3	12.0 ns	9.7	10.0 ns
	MYR B (10 µM)	2.0	1.5 ns	1.1	2.0 ns	1.5	1.3 ns	3.1	6.0 ns
	MYR N (20 µM)	1.5	0.8 ns	1.4	0.9 ns	2.0	1.2 ns	1.6	3.1 ns
Pre-cancerous patients	NC	3.6	2.8 ns	4.0	2.5 ns	2.2	2.6 ns	3.5	3.2 ns
	PC (PhIP 50 µM)	9.0	10.2 ns	15.8	13.2 ns	6.2	5.6 ns	9.6	11.0 ns
	MYR B (10 µM)	2.5	3.4 ns	6.9	3.8 ns	2.6	3.3 ns	3.9	4.5 ns
	MYR N (20 µM)	2.3	1.7 ns	4.0	2.9 ns	1.0	1.8 ns	2.1	3.1 ns

Confounding factors such as age, ethnicity, gender and smoking were analysed using the Comet assay and results demonstrated that various chemicals used in this study had no significantly different effects on either of the comparison group. Comparison was made between the variations within the confounding factor

*ns* not significant

#### Activation of the P53 and ATR signalling pathways by myricetin bulk and nanoparticles following PhIP-induced oxidative stress

**Fig. 5:**
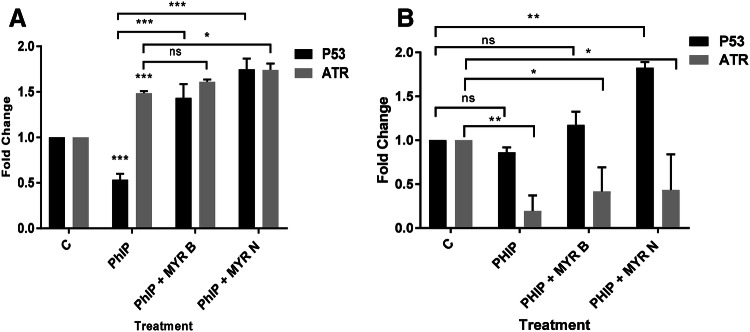
The expression of ATR and P53 mRNA in lymphocyte from healthy individuals (**a**) and pre-cancerous patients (**b**) after treating with PhIP (100 µM), MYR B (10 µM) with PhIP and MYR N (20 µM) supplemented with PhIP. GAPDH was used as an internal control gene. Gene expression analysis was performed on lymphocytes after 24-h treatment. Values are the means of three independent experiments, and the error bars represent SDs. (*P < 0.01, **P < 0.01, ***P < 0.001, *ns* not significant). Horizontal lines on the graph represent the difference between the groups. Data were compared against the control (**c**). All data were normalised against GAPDH reference gene

Built on earlier results, we found that MYR B and MYR N have shown protective effects against PhIP-induced DNA damage in lymphocytes from healthy individuals and pre-cancerous patients. To identify the molecular mechanism involved in this effect, we studied the influences of PhIP and then myricetin co-supplementation with PhIP on the gene expression levels of P53, a tumour-suppressor multi-functional gene and ATR kinase mRNA in lymphocytes. The results (Fig. 5) have shown that in healthy lymphocytes, PhIP treatment significantly decreased the P53 gene expression to 0.5-fold, however, this was significantly up-regulated upon supplementation with MYR B to 1.4-fold and with MYR N to a 1.75-fold increase. In lymphocytes from the patient group the P53 was slightly down-regulated with PhIP treatment, whereas significantly up-regulated by MYR N co-supplementation (*P* < 0.01). PhIP has shown different effects on gene expression levels of ATR in lymphocytes from healthy individuals to those from pre-cancerous patients. The ATR gene was significantly (*P* < 0.001) up-regulated by PhIP in healthy lymphocytes and it was further enhanced by myricetin supplementation where MYR N has shown *P* < 0.001 significance. However, PhIP has demonstrated reverse effects on ATR expression in lymphocytes from the patient group. It significantly down-regulated the ATR gene levels (*P* < 0.01) which were significantly up-regulated by myricetin addition. These results indicate that protective effects caused by myricetin on PhIP-induced damage might be dependent on the tumour-suppression activity of the P53 gene. The mechanisms involved in diverse ATR regulation by PhIP in lymphocytes from healthy individuals compared to those from pre-cancerous patients are not fully understood.

## Discussion

This study focused on the effects of the food mutagen, PhIP in lymphocytes from pre-cancerous patients compared to those from healthy individuals and the modulating effects of myricetin against PhIP-induced damage. PhIP has previously been shown to be genotoxic producing DNA adducts (Brown et al. 2001) and contributing towards the formation of dietary cancers (Cheung et al. 2011; Voutsinas et al. 2013). First, we demonstrated that PhIP induces significant levels of DNA damage and strand breaks in lymphocytes from healthy and patient groups, determined by the Comet and the micronucleus assays (*P* < 0.001), supporting previous studies (Mimmler et al. 2016; Buonarati et al. 1990; Boobis et al. 1994). Results from the micronucleus assay have shown a significant induction of MNi formation in BiNC after treatment with PhIP alone (*P* < 0.001). A MN formed in BiNC only depicts the damage caused after the treatment, hence reducing the probability of scoring the pre-existing damage (Magdelenova et al. 2012). In micronucleus assay, we had to rely on results deducted from 1000 cells scored per treatment group due to manual scoring. However, in future, automated micronucleus scoring techniques could be applied to score a much greater number of cells making the effect more apparent.

Our results confirm that PhIP being a genotoxic agent in the absence of toxicity causes significant DNA damage in lymphocytes from healthy individuals and those from pre-cancerous patients. However, upon treatment with myricetin (MYR B 10 µM and MYR N 20 µM), PhIP-induced damage was reduced to a substantial level presented by both the Comet and cytokinesis-block micronucleus assays (Figs. 3, 4 and Table 3). Both the control lymphocytes and patient lymphocytes showed high sensitivity to PhIP in the Comet assay. Lymphocytes from the pre-cancerous patients had an increased level of basal damage due to the disease condition. Confounding factors (age, ethnicity, gender etc.) of both investigative groups were best possibly matched and their effects on DNA damage were determined. There was no relationship established between any of the confounders and the DNA damage in healthy and patient groups (Table 4).

It is believed that food mutagens damage the DNA by producing ROS and flavonoids act in an anti-oxidant manner to reduce this damage (Kurzawa et al. 2012). Therefore, DNA damage caused by PhIP in lymphocytes from both investigative groups could possibly be because of dual mechanisms: CYP1A2-induced or ROS-induced genotoxicity.

Previous studies have shown that the apical DDR kinases such as ATR and ATM can be directly activated by DNA adducts apart from the replication-dependent stimulation (Choi et al. 2007, 2009; Kemp et al. 2011). Based on our results from the Comet assay, we established that PhIP induces strand breaks and that myricetin protects against their induction. Hence, to understand the DDR elicited by PhIP and myricetin, we investigated the gene expression levels of ATR kinase and the tumour-suppressor gene P53 in lymphocytes from healthy individuals and pre-cancerous patients. Similar patterns of results were obtained for the P53 gene in lymphocytes from both groups. P53 was down-regulated upon PhIP treatment, whereas MYR B and MYR N supplementation had shown significant attenuation of PhIP-triggered effects and increased the expression of P53 to substantial levels.

However, PhIP has shown diverse effects on the ATR kinase activity in both investigative groups. It has significantly increased the ATR gene regulation in healthy lymphocytes which was further enhanced by myricetin supplementation (*P* < 0.001). This was in agreement with previous studies that upon provoking replication stress, PhIP activates ATR–CHK1 pathway in V79 CS cells (Mimmler et al. 2016). These results propose that myricetin may protect against the mutagenicity caused by PhIP in healthy lymphocytes by triggering the activation of ATR in P53-mediated DDR pathway. Hence, contribute towards the survival path by initiating repair mechanism.

On the other hand, PhIP significantly (*P* < 0.01) down-regulated the ATR kinase activity, in lymphocytes from the patient group. However, myricetin effectively weakened the effects of PhIP and significantly increased ATR regulation. Since the ATR inhibition and increased sensitivity caused by PhIP in patient lymphocytes and the protection shown by myricetin were both dependent on the P53 pathway. This suggests that myricetin could potentially induce apoptosis in PhIP-treated lymphocytes from pre-cancerous patients. Also, the protection depicted by myricetin against PhIP-induced damage may be attributed to its anti-tumour activity by stimulating the levels of the P53-tumour-suppressor gene.

The overall findings from the current study confirm that myricetin is effectively able to prevent the DNA of lymphocytes from healthy and pre-cancerous MGUS patients from PhIP-induced DNA damage. This could possibly be made clinically applicable by maintaining the plasma concentrations of myricetin through regular intake.
